# A Very Rare Cause of Pleuritic Chest Pain: Bilateral Pleuritis as a First Sign of Familial Mediterranean Fever

**DOI:** 10.1155/2013/315751

**Published:** 2013-01-17

**Authors:** Sevket Ozkaya, Saliha E. Butun, Serhat Findik, Atilla Atici, Adem Dirican

**Affiliations:** ^1^Department of Pulmonary Medicine, Samsun Medicalpark Hospital, Samsun, Turkey; ^2^Department of Pulmonary Medicine, Samsun Chest Diseases and Thoracic Surgery Hospital, Samsun, Turkey; ^3^Department of Pulmonary Medicine, Faculty of Medicine, Ondokuz Mayis University, Kurupelit, Samsun, Turkey

## Abstract

The familial Mediterranean fever (FMF), also called recurrent polyserositis, is characterized by reccurrent episodes of serositis at pleura, peritoneum, and synovial membrane and fever. We present a patient with recurrent bilateral pleural effusion due to serositis attacks as a first sign of FMF. A 59-year-old Turkish man suffered from recurrent pleuritic chest pain due to pleural effusion and atelectasis. The etiology was not found, and his symptoms were spontaneously recovered during several weeks. The pleuritic chest pain was associated with abdominal pain in the last attack. The gene mutation analysis revealed the homozygosity of FMF (F479L) gene mutation in both our patient and his grandchild. After the colchicine treatment, the attack has not developed. In conclusion, recurrent pleural effusion and pleuritic chest pain may be the first signs of the FMF.

## 1. Introduction 

The familial Mediterranean fever (FMF), an autosomal recessive condition, affects more than one hundred thousand people worldwide and, as such, is the most common of the hereditary periodic fevers [1]. The FMF has affected mainly Mediterranean populations including non-Ashkenazi Jews, Arabs, Turks, and Armenians. It is characterized chiefly by short and periodic attacks of fever and serositis involving the pleura, peritoneum, synovial membrane, and tunica vaginalis [2]. Pulmonary involvements of FMF because of inflammation of pleura were reported by 30–40% of patients. They are usually present with unilateral pleuritis and fever [3, 4]. We present a patient with recurrent bilateral pleural effusion due to serositis attacks as a first sign of FMF. 

## 2. Case Presentation

**Figure 1:**
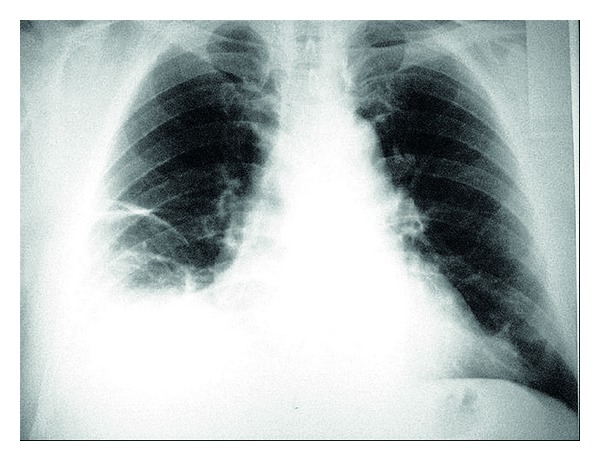
Chest radiography showing pleural effusion with linear atelectasis on the right lung.

A 59-year-old Turkish man was admitted to the hospital with pleuritic chest pain on right hemithorax, dyspnea, cough, and fever (38.5°C). Physical examination showed the decreased breath sound and pleural frotman with auscultation. The chest radiography also showed pleural effusion with linear atelectasis on right side lung (Figure 1). 

**Figure 2:**
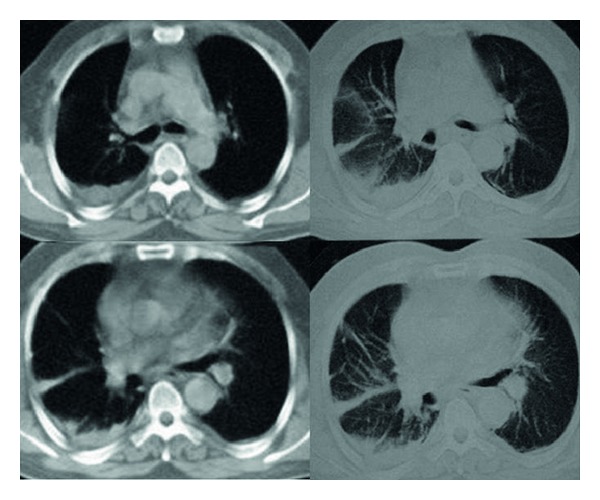
Thorax computed tomography showing the bilateral pleural effusion and linear atelectasis.

Thorax CT was performed and this showed more right sided bilateral pleural effusion and linear atelectasis (Figure 2). 

**Figure 3:**
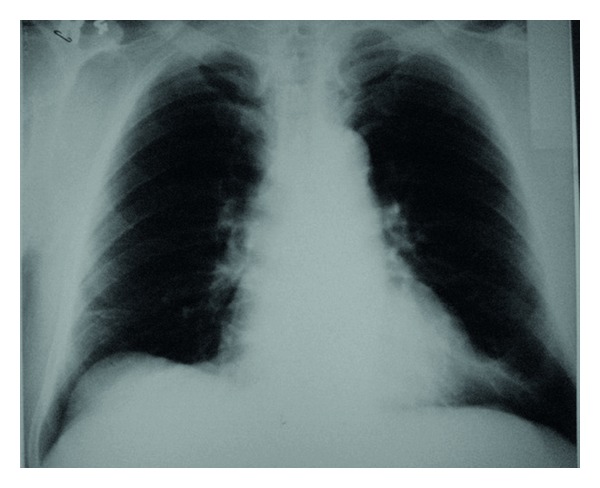
Northwest radiography showing the normal chest radiograph after the treatment.

Laboratory findings demonstrated the leukocytosis (favor to polymorphonuclear cell) and increased erythrocyte sedimentation rate (69 mm/h). The patient was treated with antibiotic (cefuroxime and clarithromycin) for pneumonia and pleuritis but did not improve. The lung ventilation-perfusion scintigraphy was performed for pulmonary thromboembolism, and it defined the match perfussion defect. The complaints of patient were recovered by reducing after several weeks. After the first attack, he suffered from recurrent symptoms as in first attack within four years. In 2008, the patient was admitted to hospital with chest and abdominal pain. Chest radiography showed pleural effusion and atelectasis. When we evaluated the findings of patient comparing with previous findings, these symptoms may be caused from FMF. Colchicine was started for treatment as dose of 1 mg/day. His symptoms were improved. Colchicine dose was increased to 1.5 mg/day. After the treatment, attack has not developed (Figure 3).

Family history was asked to confirm the FMF. We learned that his grandchild has recurrent abdominal pain and suspected for FMF. MEFV (the gene responsible for FMF) gene mutation was studied to confirm the diagnosis. The gene mutation analysis revealed the homozygosity of FMF (F479L) gene mutation in both our patient and his grandchild.

## 3. Discussion

FMF, also called reccurrent polyserositis, was characterized with reccurrent episodes of serositis at pleura, peritoneum, and synovial membrane and fever [5, 6]. The initial attack usually occurs before twenty years old and is typically dominated by peritoneal symptoms and signs. The initial attack is characterized by pleuritic chest pain and fever in fewer than 10% of patients like our case, but approximately 40% have an attack of febrile pleurisy during the course of their disease. There were no symptoms except pleuritic chest pain in our patient. Livneh and Langevitz described the diagnostic criteria of FMF [7]. According to these criteria, unilateral pleuritis is one of the major criteria of FMF. But, bilateral pleuritic attack was the first sign of our patients. According to our knowledge, this case is the first case report with bilateral pleuritis and the first sign of FMF. Chest radiographs during the acute pleuritic attacks show the elevation of the ipsilateral diaphragm and frequently small pleural effusions. In our patient, the chest radiography showed unilateral pleural effusion, but computed tomography revealed the bilateral pleural effusion. The attacks are reccurrent, with irregular intervals of days to months between the attacks. Because the administration of colchicine decreases the frequency of the attacks [8], there were no attacks in our patients after the colchicine treatment. 

In conclusion, recurrent pleural effusion and pleuritic chest pain may be the first signs of the FMF. Also, FMF attacks may be one of the reasons of the bilateral plural effusions. For patients who have unexplained bilateral pleural effusion and chest pain with fever, FMF should be keep in mind, especially in the Mediterranean region.
